# Polyphenolic Profiling, Antioxidant, and Antimicrobial Activities Revealed the Quality and Adaptive Behavior of Viola Species, a Dietary Spice in the Himalayas

**DOI:** 10.3390/molecules27123867

**Published:** 2022-06-16

**Authors:** Rishabh Kaundal, Manish Kumar, Subhash Kumar, Dharam Singh, Dinesh Kumar

**Affiliations:** 1 Chemical Technology Division, CSIR-Institute of Himalayan Bioresource Technology, Palampur 176061, Himachal Pradesh, India; rishabh.ihbt19a@acsir.res.in (R.K.); manish9644@gmail.com (M.K.); 2 Academy of Scientific and Innovative Research, Ghaziabad 201002, Uttar Pradesh, India; subhashkumar136@gmail.com (S.K.); dharamsingh@ihbt.res.in (D.S.); 3 Biotechnology Division, CSIR-Institute of Himalayan Bioresource Technology, Palampur 176061, Himachal Pradesh, India

**Keywords:** Viola species Banksha, adaptation, polyphenols, antioxidants, antibacterial

## Abstract

Background: Himalayan Viola species (Banksha) are traditionally important herbs with versatile therapeutic benefits such as antitussive, analgesic, antipyretic, antimalarial, anti-inflammatory, and anticancerous ones. The current investigation was focused on exploring polyphenolic profiles, antioxidant, and antimicrobial potentials of wild viola species at 15 gradient locations (375–1829 m). Methods: Morphological, physiochemical, and proximate analyses were carried out as per WHO guidelines for plant drug standardization. Total polyphenolic and flavonoid content were carried out using gallic acid and rutin equivalent. UPLC-DAD was used to profile the targeted polyphenols (gallic acid, vanillic acid, syringic acid, *p*-coumaric acid, ferulic acid, rutin, quercetin, luteolin, caffeic acid, and epicatechin). Similarly, all samples were screened for antioxidant and antimicrobial activity. Statistical analysis was used to correlate polyphenolic and targeted activities to assess Viola species adaptation behavior patterns. Results: *Viola canescens* (*V. canescens*) and *Viola pilosa* (*V. pilosa*) were found abundantly at their respective sites. Among flowers and leaves, flowers of *V. canescens* and *V. pilosa* showed higher total polyphenolic and flavonoid content (51.4 ± 1.13 mg GAE/g and 65.05 ± 0.85 mg RE/g, and 33.26 ± 0.62 mg GAE/g and 36.10 ± 1.41 mg RE/g, respectively). Furthermore, UPLC-DAD showed the uppermost content of *p*-coumaric acid in flowers and ferulic acid in leaves, while rutin was significant in both the tissues. Conclusions: The adaptive behavior of Viola species showed variability in morphological characters with the altitudes, while targeted polyphenols and activities were significant at mid-altitudes. This research helps in the selection of right chemotype for agrotechnological interventions and the development of nutraceutical products.

## 1. Introduction

From ancient times, natural products have long been recognized as an abundant source of therapeutic medicines [1]. However, the use of traditional drugs is limited due to lack of authenticity and quality [2]. The use of advanced sophisticated analytical tools enabled us to fill this gap and correlate the pharmaceutical properties of traditional medicines with their bioactive products [3]. On the other hand, increasing population, urbanization, and unrestricted collection of raw material from wild plants results in over-exploitation of natural flora [4]. Some of the natural calamities also diminish the species from their habitats. Hence, the scientific intervention and management of traditional medicinal plant resources have become a matter of urgency. The Indian subcontinent is one of the mega biodiversity centers of the world’s biodiversity wealth. Out of 17,000 species of higher plants reported to occur in India only, 7500 are known to have medicinal properties [5,6]. Still, several medicinally important plants available are not exploited for their chemical and therapeutic potential [7]. These plants are locally used by the villagers or communities for their needs based on their experiences and traditional knowledge without awareness among them for their conservation. Moreover, some of the medicinal plants adapted to different environmental conditions and produce specific metabolites to fight the biotic and abiotic stresses [8]. During these processes, the quality of the raw material may change and need to be assessed.

The family Violaceae, consisting of around 800 species having more than 25 genera, are distributed throughout the world [9]. Viola species (sweet violet and Banafshe in Farsi) are found in the temperate northern hemisphere (Iran, Iraq, Andes, Australia, Hawaii, Malaysia, China, Nepal, Sri Lanka, Pakistan, and India). In India, it is distributed in the Himalayan range from Jammu and Kashmir to Meghalaya, including Himachal Pradesh [10] and Uttarakhand. Previously, the therapeutic potential of Viola species (*V. odorata* [11], *V. canescens* [12], *V. cinerea* [13], *V. Serpens* [14], and *V. pilosa* [15]) were documented. These species have demulcent, astringent carminative, diuretic, antipyretic, anti-asthmatic, purgative, diaphoretic, and anticancerous properties. In traditional and folk practices, these species are used against stomach acidity, eczema, epilepsy, rheumatism, jaundice, and respiratory problems [12,14,16]. Viola species contains alkaloid, glycoside, flavonoids, terpenes, saponins, methyl salicylate, amino acids, essential oil, mucilage, and vitamin C etc. [17,18,19,20]. Thus, huge demand for Viola at the national and international level created the interest of scientists in its conservation, domestication, and quality control through agrotechnological and quality-control interventions. In respect, Viola species (Violaceae) of temperate Himalaya have been focused on to assess their adaptation pattern, chemical ecology, and therapeutic potentials.

## 2. Results and Discussions

**Figure 1:**
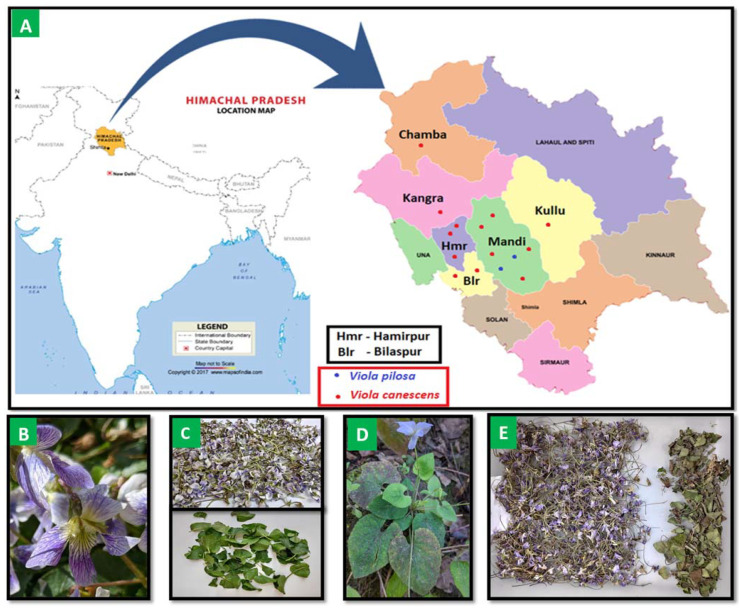
(**A**) Viola species collected from the study area (altitude of 375 to 1829 m) of Himachal Pradesh, India (**B**). *V.canescens* (**C**). Flowers and leaves of *V.canescens* (**D**). V. pilosa (**E**). Flowers and leaves of *V. pilosa*.

Viola species are important spices of therapeutic benefits particularly to treat fever, cough, and respiratory problems. Traditionally, and in tribes of the Himalayas around the world, Viola is used in home remedies and is very popular in the Indian system of medicine. Two Viola species (*V. canescens* and *V. pilosa)* were observed at gradient altitudes (altitude of 375 to 1829 m; 15 locations). *V. canescens* were noticed majorly throughout the Himalaya while *V. pilosa* were observed only in midrange (750–1300 m). Some of the earlier reports highlighted the presence of *V. serpens* at nearby altitudinal locations such as Ghumarwin, Awahdevi, and Patta [14], but these species were not noticed in the current study areas. Thus, further elaborative study will be conducted to cover the whole Viola vicinity of the Himalayas in upcoming research. The study area, presence of Viola species, and collected materials are represented in Figure 1, Table 1, and Supplementary Table S1. It was observed that the forest-shady locations and hill roadsides with moisture at low and high altitudes showed the presence of *V. canescens*, while high mountain pasture with sun phase at mid-altitudes showed *V. pilosa*. Morphological variations were also observed in the Viola species at various altitudes. The color of the flower varies in the genus, differentiating from violet through various shades of blue, yellow, white, and cream, while some types are bi and tricoloured [15,21,22]. In the study areas, *V. canescens* were observed in violet color flowers with petals while leaves are alternate, stipules, and persistent, whereas flowers of *V. pilosa* were noticed as whitish in color with alternate leaves.

**Figure 2:**
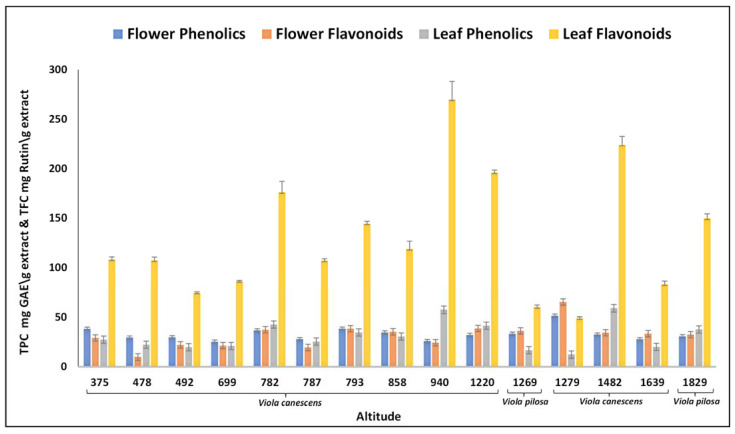
Total phenolic and total flavonoid contents accumulation in Viola species at gradient altitudes. GAE—gallic acid equivalent.

The preliminary phytochemical analysis of Viola species revealed the presence of carbohydrates, proteins, lipids, tannins, steroids, terpenoids, alkaloids, saponins, phenols, and flavonoids. The flavonoids and phenolics showed dominancy in preliminary tests. Thus, total phenolics content (TPC) and total flavonoid content (TFC) were estimated in the samples of trans-Himalayas to monitor polyphenolic regulations at gradient altitudes. The results were derived from a calibration curve (flowers: y = 0.014x − 0.0592, r^2^ = 0.99 and leaves: 0.0109x − 0.028, r^2^ = 0.99) of gallic acid (0–100 µg/mL) and expressed in gallic acid equivalents (GAE) per gram dry extract weight for phenolics and for flavonoids; the results were derived from the calibration curve (flowers: y = 0.0034x − 0.0058, r^2^= 0.98 and leaves: y = 0.0014x − 0.0087, r^2^ = 0.98) of rutin (0–100 µg/mL) and expressed in rutin equivalent per gram dry extract weight. The highest phenolic content in *V. canescens* leaves was recorded (58.75 ± 1.78 mg GAE/g) at an altitude of 1482 m and in flowers (51.4 ± 1.13 mg GAE/g) at an altitude of 1279 m. The TFC was highest (65.05 ± 0.85 mg RE/g) at 1279 m in the case of flower and at 940 m in leaves (270.02 ± 18.40 mg RE/g). The TPC and TFC were observed higher in the case of *V. canescens* as compared with *V. pilosa* (Figure 2). All the locations exhibited significant TPC and TFC with variable amounts. The content was initially found to be decreased with the increase in altitudes and then increased to some extent at middle altitude. Additional increase in altitude again decreased the content. This fluctuation in the TPC and TFC might be due to the environmental effects or stressful conditions at high altitude [23]. A clear fluctuation can be seen at gradient elevations as depicted in Table 1 and Figure 2. Furthermore, trace elements are mineral nutrients required by the plants to perform vital metabolic processes. Besides biological functions, these elements are utilized by the people of the current world towards the treatments of metabolic disorders. Around forty elements found to be essential to the living systems [24] and four heavy metals (As. Pb. Hg and Cd) were found toxic beyond certain limits. These toxic heavy metals were not found in fifteen locations. Nine trace elements (Fe, Zn, Mg, Mn, Cu, Ni, Ca, Na, and K) were found in the leaves and flowers of Viola species. These elements were varied at gradient altitudes and the trend of their presence revealed a wave-like pattern. The elemental variations are depicted in Table 1, Supplementary Figures S1 and S2.

### 2.1. Antioxidant Activity

Plant antioxidants decreased absorbance, which showed the reduction capability of DPPH and ABTS radicals. *V. canescens* showed better antioxidant potential than *V. Pilosa*, and flowers have more antioxidants than leaves. In the DPPH assay, low altitude flowers (375 m) showed the highest scavenging activity (IC_50_ 0.24 ± 0.01, mg/mL), but in the case of leaves it was at altitudes of 1482 m (IC_50_ 0.49 ± 0.01, mg/mL). Whereas in ABTS assay, the highest potential of flowers was shown at altitudes of 787 m and 1220 m (IC_50_ 0.06 and 0.07 ± 0.002, mg/mL, respectively), and leaves showed at an altitude of 1482 m (IC_50_ 0.23 ± 0.12, mg/mL). The alterations of antioxidant activities at varied altitudes are shown in Table 1. The variation in activity might be due to the environmental factors and presence of antioxidant metabolites. Among all the altitudinal samples, 1482 m showed significant results of flower and leaf and appears to be a favorable location for *V. canescens* cultivation, while 1829 m is suitable for *V. pilosa* cultivation (Supplementary Figure S3).

### 2.2. Polyphenols Determination

**Figure 3:**
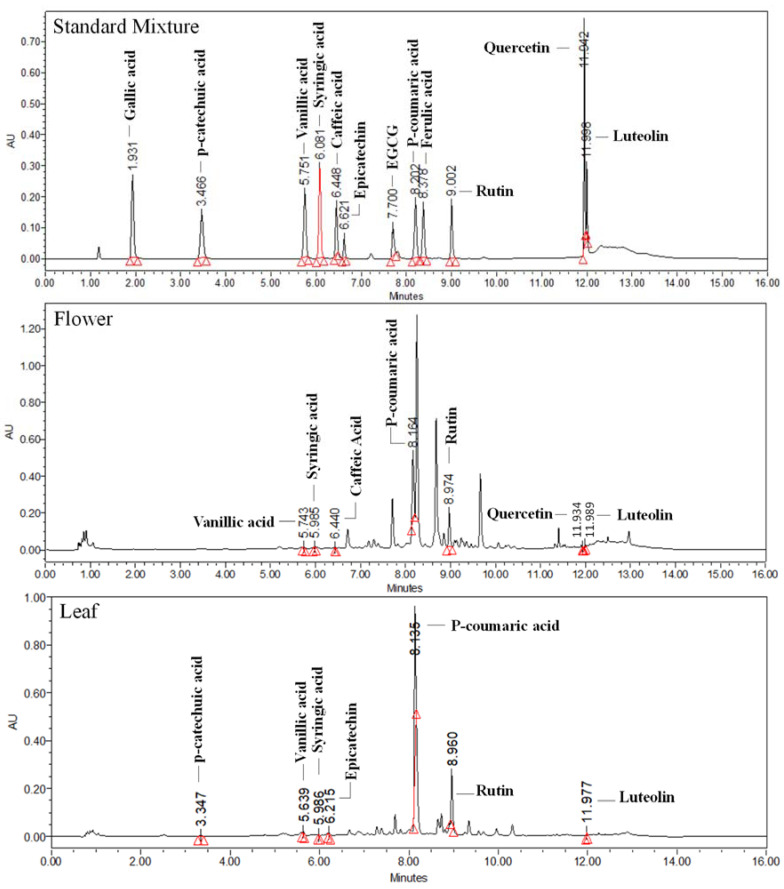
Representative chromatograms of reference standard mixture, flowers, and leaves samples at 270 nm.

**Scheme 1:**
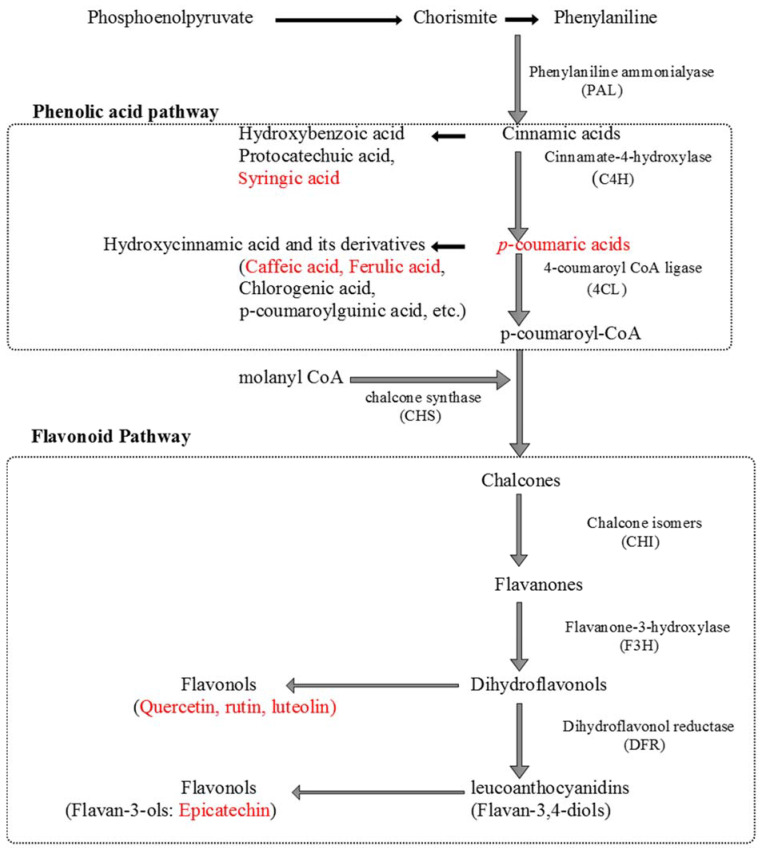
Biosynthesis of the targeted polyphenols.

The phenolics and flavonoids were identified and quantified in Viola species collected from gradient altitudinal locations. Three flavonoids (quercetin, luteolin, and rutin) and six phenolic acids (epicatechin, vanillic acid, *p*-coumaric acid, ferulic acid, syringic acid, and caffeic acid) were identified in the flowers collected from various locations. Polyphenols are majorly synthesized by the shikimic acid pathway. They are helpful in plant growth and have antioxidant and anti-inflammatory activities [25,26]. The *p*-coumaric acid was found higher in flowers (5.02 ± 0.05–23.406 ± 1.77 mg/g) of all altitudes, and ferulic acid (1.78 ± 0.05–14.97 ± 1.2 mg/g) in leaves, as compared with other targeted metabolites. It was also observed that syringic acid and quercetin were not present in flowers, except at 1279 m, while quercetin was absent in leaves. Furthermore, caffeic acid was not quantifiable in leaves of all altitudes, except 1220 and 1482 m. Rutin (Vit. P), a bioflavonoid, was significant in flowers and leaves (0.19 ± 0.01–4.68 ± 0.00, 0.23 ± 0.00–10.63 ± 0.7 mg/g, respectively), and luteolin (0.43 ± 0.03 mg/g) in leaves. The variations of targeted metabolites in flowers and leaves at gradient altitudes were observed (Table 2; Figure 3). The UPLC-DAD chromatograms of both the flowers and leaves samples were depicted in the Supplementary Figure S4a,b, while schematic biosynthesis of the targeted metabolites was also depicted in Scheme 1.

### 2.3. Antimicrobial Activity

Viola species collected from the different areas were assessed for antibacterial potential against pathogenic bacteria, i.e., Gram-positive (*B.* *subtilis* and *S*. *aureus*) and Gram-negative (*S. typhimurium* and *E. coli*). The zone of inhibition was depicted in Table 3, Supplementary Table S2 and Supplementary Figure S5. The Viola flower and leaves extracts were potentially inhibiting the bacterial growth. The flower samples DKRV7 (793 m), DKRV9 (940 m), DKRV12 (1279 m), and DKRV13 (1482 m) showed a maximum 5.0 mm zone of inhibition (radius, mm) against B. subtilis and 1.0, 2.0, 2.5, 2.5 mm against S. aureus at a concentration of 6 mg crude flower extract. In the case of leaves extract, the most effective samples were from an altitude of DKRL1 (375 m), DKRL13 (1482 m), and DKRL14 (1639 m), which showed a 4.0 mm zone of inhibition against B. subtilis and 3.0, 4.0, and 4.0 mm against S. aureus. Gram-positive bacteria exhibited a zone of inhibition in all extracts, while Gram-negative bacteria displayed no zone of inhibition. The most effective leaf and flower extracts’ minimum inhibitory concentrations (MICs) were also evaluated. MICs at 5 mg concentration showed a zone of inhibition in crude extract of all the selected leaf and flower samples. The flower extracts showed a wide zone of inhibition as compared with leaf extracts. In this study, it was also observed that the zone of inhibition is directly proportional to the altitude in the case of the collected flower samples. In a few cases, activity was dropped or decreased, which may be because of the environmental conditions of those altitudes (Table 3). In the leaf extracts, the zone of inhibition was found in all the selected samples, but there is no such correlation with altitude. The Viola species were previously reported for their strong antimicrobial agent, which may be due to their phenolics, flavonoids, alkaloids, cyclotide, and saponins [27,28]. Cyclotides derived from V. *odorata* exhibited antibacterial efficacy against pathogenic bacteria such as E. coli, P. aeroginosa, and S. aureus [27]. The aerial parts of *V. odorata* used as an aqueous extract exhibited antimicrobial activity against *S.aureus*, *B. subtilis*, *E. coli*, and *S.* *flexneri* [29]. Furthermore, the antimicrobial activity of Viola was also observed against the respiratory tract pathogen [30].

### 2.4. Adaptive, Correlation, Similarities, and Variations Insights of Viola Species at Gradient Altitudes

**Figure 4:**
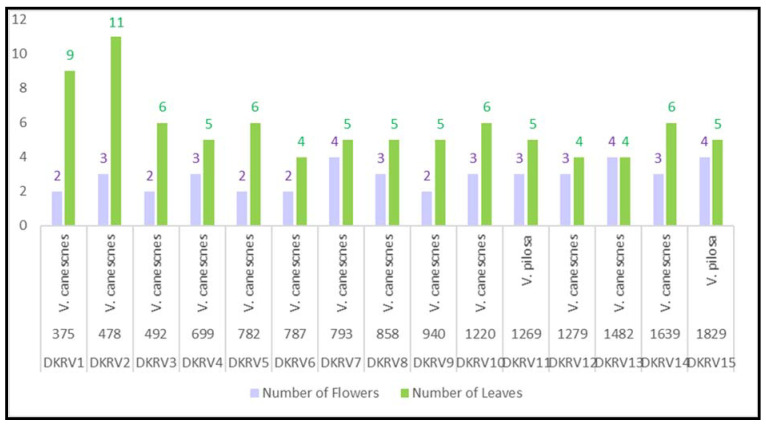
Leaf number variation at gradient altitudes of Viola species.

The adaptive parameters, such as morphological characteristics, extractives, chemical representations, phenolic, flavonoids, antioxidant, and antimicrobial insights at gradient elevations, were correlated through statistical analysis. It was observed that *V. canescens* was dominant in most of the locations in the alpine Himalayan. The *V. pilosa* was found only at two locations among fifteen in the studied areas. Furthermore, leaves were found decreased with an increased altitude, while flowers did not have much difference (Figure 4).

**Figure 5:**
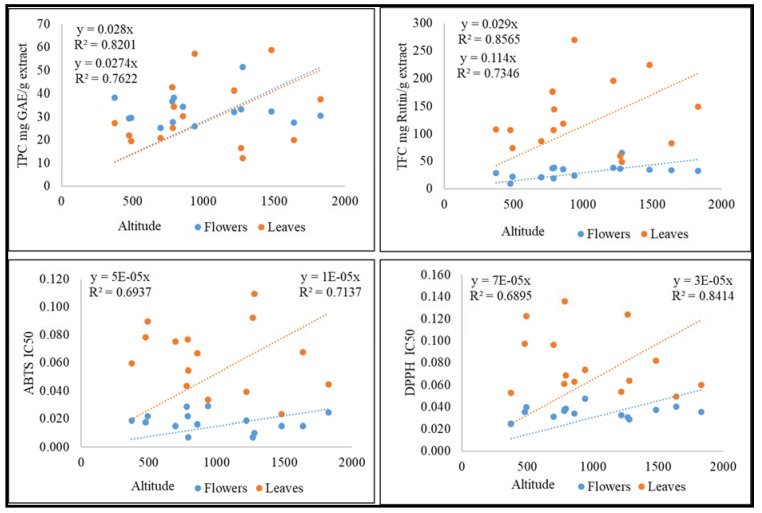
Correlation of TPC, TFC, ABTS, and DPPH with altitudes.

The extractive yield in 70% ethanol was found significant between the range 25–45% (SI-1), and phytochemical analysis of extracts represents the chemical compounds as present in the initial preliminary studies. Furthermore, leaves and flowers showed a significant amount of polyphenols, flavonoids, and antioxidant activity, which were correlated with the altitudes and correlation coefficient depicted in Figure 5. Leaves contain more polyphenols, but the antioxidant activity was found to be highest in flowers. This might be due to the contribution of other classes of molecules present in flowers. Additional inter-relationship between the antimicrobial activity of leaves and flowers was noticed. An increase in the antimicrobial activity of flowers decreases the antimicrobial potential of leaves and vice versa. The accumulation of metabolites and bioactivities at specific altitudes and locations might be due to the requirements of the environment for survivability. The adaptation to specific environments alters the chemistry and structure of the species. Hence, Viola species showed different trends for both flowers and leaves at varying altitudes.

**Figure 6:**
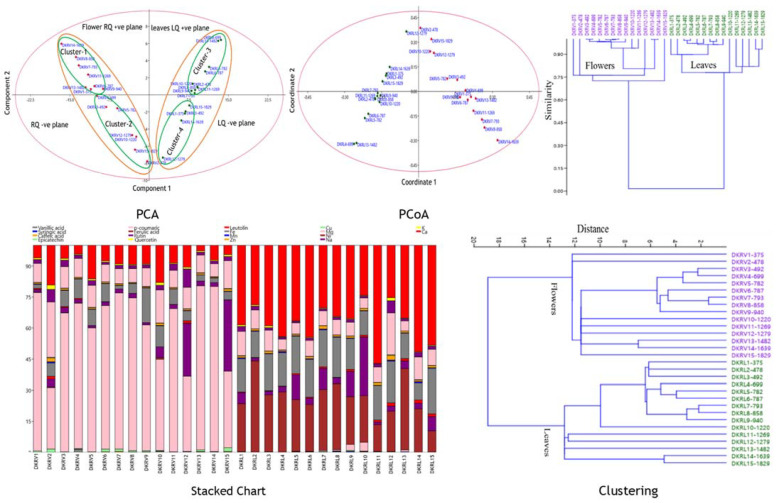
PCA, PCoA, stacked charts, and matrix plot of leaf and flower (TPC, TFC, UPLC-Polyphenols, and antioxidant activity) at gradient altitude. PCA: principal component analysis; PCoA: principal coordinate analysis; HCA: hierarchical clustering analysis. DKRV1 to DKRV15: flowers samples; DKRL1to DKRL15: leaves samples.

Furthermore, the quantitative data (TPC, TFC, targeted polyphenols, and antioxidant activity) obtained from the Viola species were analyzed with multivariate statistical techniques, which revealed the similarities, discriminations, and correlations among the samples collected from varying altitudes. It was observed that leaves or flowers at gradient altitudes have qualitative similarities and quantitative differences. The targeted components showed associations and variations among the gradient altitudinal samples. The dominancy of *p*-coumaric acid and rutin was observed in flowers, while ferulic acid, luteolin, and quercetin in leaves among the targeted polyphenols was observed (Figure 6). The statistical analysis (PCA, PCoA, stacked charts, and matrix plot) visualized the clear differences among the leaves and flowers of Viola species. The results of the principal component analysis (PCA and PCoA) showed that the samples were different and observed in quadrants of the score plot (Figure 6). Both parts lie in the right (flowers) and left quadrants, which further divided into positive and negative planes of the respective quadrants. The study revealed that fifteen altitudinal samples were representatives of chemotypes and were grouped into four distinct clusters (flowers-2 cluster and leaves-2 cluster) in PCA and PCoA. Cluster-3 was the largest cluster, comprising 10 chemotypes, followed by cluster-1 (8 chemotypes), cluster-2 (6 chemotypes), and cluster 4 (5 chemotypes). The cluster sets were positively and negatively influenced by their metabolite content. Cluster-1 and 3 were positively correlated and clusters-2 and 4 were negatively correlated with metabolites and found environmentally adopted nutritionally enriched chemotypes (Figure 6). The eigenvalues of the measured metabolites in samples of different locations observed the variation between the principal component (PC) axes. The PCA of the PC samples’ axes along with the major percent variation PC1 are: eigenvalue (%): 93.58 (79.82), 14.30 (12.20), 4.38 (3.74), 3.16 (2.70), 1.06 (0.90), 0.65 (0.56), and others were <0.5 (0.05), while from the coordinate PCoA: PCo1; eigenvalue (%): 2714.1 (79.82), 414.89 (12.20), 127.26 (3.74), 91.71 (2.69), 30.64 (0.90), 19.01 (0.55), 1.28 (0.04), and others were <1 (0.05). The hierarchical clustering analysis showed the association of flowers and leaves. Multivariate statistical analysis deciphered the equipotent potential of leaves and flowers. Stacked plot (Figure 6) showed the clear qualitative and quantitative similarities, correlations, and variations among the different samples collected from the vicinity of the western Himalaya of Himachal Pradesh, India.

## 3. Experimental

### 3.1. Chemicals

All the chemicals used were of analytical grade. The chemicals such as 1,1-Diphenyl-2-picrylhydrazyl (DPPH), ascorbic acid, 2,4,6-tri (2-pyridyl)-s-triazine (TPTZ), ABTS radical + [(2,2′-Azino-bis (3-ethylbenzothiazoline-6-sulfonic acid) diammonium salt], gallic acid, vanillic acid, syringic acid, *p*-coumaric acid, ferulic acid, rutin, quercetin, luteolin, caffeic acid, epicatechin, aluminium trichloride, potassium acetate, anhydrous sodium carbonate, sodium acetate, ferric chloride hexahydrate, folin-ciocalteu reagent, dragendorff’s reagent, mercuric chloride, potassium iodide, chloroform, ammonia, glacial acetic acid, iodine, ethanol, methanol, hydrochloric acid, sulfuric acid, and sodium hydroxide were purchased from Merck-Sigma-Aldrich, India.

### 3.2. Collection and Authentication

The plant samples of Viola species were collected from gradient habitats (altitude of 375 to 1829 m; 15 locations) of the northwestern Himalayas of Himachal Pradesh, India. Above-ground parts of the plants were collected during the flowering stage (February–April, 2019). The plant specimens were collected from the deep vicinity of the Viola (5 m × 5 m plots) × 10 = 10 samples from each location. These samples were morphologically validated and submitted for authentication at the institute’s plant authentication department (Department of Environmental Technology, CSIR-IHBT, Palampur, H.P. India). The plant specimens were identified as *V. canescens* and *V. pilosa* at gradient altitudes of Himachal Pradesh, India. The voucher specimen numbers PLP16476, PLP16471, PLP16472, PLP16475, PLP16475, and PLP16474 represented *V. canescens*, while PLP16477 and PLP16473 as *V. pilosa.* Furthermore, plant materials were collected for the analyses with the following information: morphological description, phase of plant development at the time of sampling, and the specific habitat descriptions.

### 3.3. Extraction and Sample Preparation

#### Sample Preparation

The plant material was collected and cleaned, flowers and leaves were separated for analysis and dried at room temperature, further crushed into powder, and stored in an airtight glass container. The flowers and leaves were macerated for 24 h with 70% ethanol. The solvents of the extracts were evaporated on a vacuum rotatory evaporator under reduced pressure. The yields are depicted in the Supplementary Table S1.

### 3.4. Preliminary Phytochemical Analysis, Total Phenolic, and Flavonoid Contents

Various phytochemical tests were performed to identify the presence of primary and secondary metabolites in the plant extract of Viola species using the standard protocol. Furthermore, the phenolic acids and flavonoids represent the presence of polyphenols. Hence, the content of total polyphenolic and flavonoid in the different samples of Viola species at gradient elevations were analyzed as gallic acid and rutin equivalent (mg/g), as described by Sharma et al. [31,32].

### 3.5. Mineral and Trace Element Analysis

The trace elements (Na, Cu, Zn, Ca, Mn, Fe, Mg, and K) and heavy metals (Pb, Cd as toxic, and Ni, Cr as essential) were analyzed in the raw material of Viola species collected from gradient altitudes as described in the AOAC method using atomic absorption spectroscopy [33].

### 3.6. Determination of Polyphenolic Traits in Viola Samples Using UPLC-DAD Method

The identification and quantification of selected phenolic acids and flavonoids (gallic acid, vanillic acid, syringic acid, p-coumaric acid, ferulic acid, rutin, quercetin, luteolin, caffeic acid, and epicatechin) in samples were performed by Waters Acquity UPLC, H-class system. The analytical column used was the Acquity BEH C18 column (2.1 mm × 100 mm, 1.8 µm). The detection wavelength was set at 270 nm. The gradient elution system was used, mobile phase A contained 0.1% formic acid in the water, and mobile phase B was 0.1% formic acid in acetonitrile (ACN). The gradient started from 0 min at 5% B maintained till 0.3 min; the concentration of B increased to 30% from 0.3 min to 9 min, then 30% B to 70% B; 9–11 min, 50% B; from 11–12 min, 50% B, then at 12.2 min, mobile phase maintained to initial conditions, 5% B, maintained till 16 min elution was performed at a solvent flow rate of 0.30 mL/min. The targeted compounds were identified using retention time and UV spectrum (λmax). The quantification of compounds was performed by calibration curve and area under the peak. Each sample was analyzed in triplicate.

### 3.7. Antioxidant Activity

#### Free Radical Scavenging Activity

DPPH and ABTS radical scavenging activity of various samples was performed by the method described in Kumar et al., [34]. The different extracts of Viola species (1 mg/mL each) were diluted (5–200 μL for flowers and 10–100 μL; 25–250 μL for leaf ABTS and DPPH, respectively), and MeOH was added to make a total volume of 200,100 and 250 μL, respectively. In each dilution 1 mL of DPPH and 0.7 mL of ABTS, solution was mixed well and incubated at 37 °C at for 30 min in dark conditions. Additional absorbance was measured at 517 and 734 nm, respectively, in a 96-well plate using a Synergy H1 microplate reader (BioTek Instruments, Winooski, VT, USA). The ascorbic acid (1 mg/mL) in ethanol was taken as a reference standard. The standard calibration curves were prepared and IC_50_ values of samples were calculated. The experiment was repeated thrice.

### 3.8. Antimicrobial Activity

The antimicrobial activity was performed using disc diffusion method with minor modification as reported earlier [35,36]. The bacterial cultures such as the *Bacillus subtilis*121, *Staphylococcus aureus*96, *Salmonella typhimurium*733, and *Escherichia coli*43 were procured from MTCC (Microbial type culture collection), Chandigarh. Briefly, the 100 μL bacterial culture (cell density 1.5 × 10^8^ CFU/mL) was used to prepare a lawn with the aid of a sterile cotton swab on a nutrient agar plate. The nutrient agar plates were allowed to stand for bacterial culture absorption for 8–10 min. The agar diffusion wells were punched in seeded plates with the help of sterile gel puncture (6 mm). The crude samples of a plant extract with the final concentration of 6.0 mg were used in each well. The plates were incubated for 10–12 h at 37 °C and further tested for the zone of inhibition. Methanol was used as a solvent control. The zone of inhibition for different leaf and flower extracts against different bacteria were measured in millimeters for further analysis. An agar well (6 mm) with no inhibition zone was regarded as having no antimicrobial activity. All tests were conducted in triplicates. Furthermore, based on the preliminary screening, the minimum inhibitory concentration (MIC) for each bacterial sample was determined. The methanolic extract of the samples that indicated potent antimicrobial activity were further tested, and the measurement of MIC, the concentrations of 0.5, 1.0, 2.0, 3.0, 4.0, 5.0, and 6.0 mg, were used. The least concentration was observed and noted as the MIC value where the extract indicated an inhibition region. All samples were subjected to triplicate.

### 3.9. Adaptive Correlation, Similarities, and Variational Insights of Viola Species at Gradient Altitudes

The adaptive parameters such as morphological characteristics, extractives, chemical representations, phenolics, flavonoids, antioxidant, and antimicrobial insights at gradient elevations were correlated through statistical analysis (PCA, PCoA, HCA, correlations, stacked plots, etc.). The datasets of targeted metabolites were subjected to statistical analysis through Past 4.02 software.

## 4. Conclusions

Viola genus is the largest genus, containing 500 species belonging to the Violaceae family and distributed throughout the globe. Viola species are also found in the Indian continent and are commonly known as Banksha/Bansfa/Banafsa/Banfsha. The western Himalayas have one of the richest repositories of Viola and were studied earlier by several groups. Hence, the study of Viola in the northwestern Himalayas of Himachal Pradesh, India was conducted to explore its species and their chemical and therapeutic potentials. The 15 gradient altitudinal locations in Himachal Pradesh, India were surveyed, which resulted only two Viola species (*V. canescens* and *V. pilosa).* Among them, *V. canescens* was found abundant in the targeted locations, while *V. pilosa* was observed in two locations. Flowers and leaves parts of both the species were found with alterations in morphology, polyphenolics, elemental, antioxidant, and antimicrobial patterns at gradient altitudes. The targeted polyphenols, nutritional components, and activities discriminated both the parts and revealed that it could be due the environmental conditions of the respective locations. Furthermore, the overuse and uncontrolled exploitation of these plant species may make them extinct in the future. Thus, the current findings help to select the right chemotype and environment for agrotechnological interventions to promote its cultivation and conservation.

## Figures and Tables

**Table 1:** Viola species study area (Himachal Pradesh, India), Altitudes, Nutritional profiling (Total phenolic and flavonoid contents and minerals), and antioxidant potentials.

Sample Code	Sample Location	Species	Altitudes (m)	Mineral Nutrients (mg/kg)									Antioxidant Activity	
				Fe	Mn	Zn	Cu	Mg	Ni	Na	K	Ca	DPPH IC_50_ (mg/mL)	ABTS IC_50_ (mg/mL)
DKRV1	Berthin, Bilaspur	*V. canescens*	375	414.16	49.22	123.46	21.3	1878.96	0.00	391.32	155.4	1252.0	0.24 ± 0.01	0.18 ± 0.00
DKRV2	Telkar, hamirpur	*V. canescens*	478	447.44	44.20	130.98	19.46	1851.70	0.00	424.92	153.8	1325.2	0.39 ± 0.04	0.17 ± 0.02
DKRV3	Berru, Hamirpur	*V. canescens*	492	1406.58	43.68	135.74	17.6	1893.52	6.40	547.16	162.6	1137.8	0.40 ± 0.00	0.21 ± 0.02
DKRV4	Ghumarwin, Bilaspur	*V. canescens*	699	1909.76	40.50	113.00	19	1861.58	9.06	341.76	93.9	845.10	0.31 ± 0.06	0.14 ± 0.05
DKRV5	Bijni, Mandi	*V. canescens*	782	1366.32	34.48	108.40	23.16	1860.74	3.36	403.68	126.0	2817.3	0.36 ± 0.01	0.21 ± 0.01
DKRV6	Chabutra, Hamirpur	*V. canescens*	787	1938.40	38.34	129.60	22.7	1878.40	7.54	240.32	126.1	1672.4	0.39 ± 0.08	0.06 ± 0.00
DKRV7	Paddar, Mandi	*V. canescens*	793	418.80	43.86	173.22	25.02	1872.60	2.98	409.16	165.2	2290.8	0.38 ± 0.02	0.16 ± 0.00
DKRV8	Pandoh, Mandi	*V. canescens*	858	1295.88	39.50	100.42	21.3	1839.68	5.26	404.36	123.8	2564.8	0.33 ± 0.03	0.29 ± 0.02
DKRV9	Batour, Mandi	*V. canescens*	940	3840.42	54.74	105.94	22.7	2053.30	1.10	365.06	99.00	2032.0	0.47 ± 0.00	0.18 ± 0.09
DKRV10	Chauntra, Mandi	*V. canescens*	1220	1268.02	20.42	126.36	23.62	2003.28	1.10	283.74	168.3	2248.9	0.32 ± 0.05	0.07 ± 0.002
DKRV11	Kamand, Mandi	*V. pilosa*	1269	1858.66	37.82	118.68	24.56	2079.14	4.12	760.76	111.3	2132.0	0.30 ± 0.04	0.10 ± 0.00
DKRV12	Kullu	*V. canescens*	1279	863.92	39.34	123.46	21.78	1856.08	1.86	1583.76	154.0	1893.7	0.28 ± 0.04	0.15 ± 0.01
DKRV13	Chandpur, Kangra	*V. canescens*	1482	610.78	18.74	181.96	26.4	1801.26	0.00	298.48	133.8	556.86	0.37 ± 0.03	0.15 ± 0.03
DKRV14	Gulera, Chamba	*V. canescens*	1639	660.32	43.68	120.38	26.86	1840.82	0.00	380.52	131.5	2103.4	0.40 ± 0.02	0.24 ± 0.02
DKRV15	Barot, Mandi	*V. pilosa*	1829	545.76	20.58	139.58	27.8	1827.96	0.00	275.76	118.8	605.06	0.37 ± 0.02	0.28 ± 0.02
DKRL1	Berthin, Bilaspur	*V. canescens*	375	3361.24	91.56	194.10	25.48	2455.92	0.00	495.8	173.4	8095.9	0.52 ± 0.11	0.59 ± 0.30
DKRL2	Telkar, hamirpur	*V. canescens*	478	3342.66	82.86	219.90	23.62	2425.56	0.00	488.78	171.9	7640.8	0.97 ± 0.03	0.78 ± 0.39
DKRL3	Berru, Hamirpur	*V. canescens*	492	3568.7	52.40	208.54	17.6	2021.22	0.00	242.60	183.4	7861.2	1.22 ± 0.25	0.89 ± 0.45
DKRL4	Ghumarwin, Bilaspur	*V. canescens*	699	6085.36	215.78	238.96	44.46	1972.48	1.10	373.06	77.6	16824	0.96 ± 0.01	0.75 ± 0.38
DKRL5	Bijni, Mandi	*V. canescens*	782	6460.04	94.24	262.76	29.64	1953.56	0.00	261.92	86.7	13070	0.60 ± 0.01	0.43 ± 0.22
DKRL6	Chabutra, Hamirpur	*V. canescens*	787	5664.24	123.88	317.28	35.66	2366.08	0.00	377.28	109.6	13930	1.36 ± 0.04	0.76 ± 0.39
DKRL7	Paddar, Mandi	*V. canescens*	793	5049.6	122.70	448.30	22.7	2030.84	0.00	301.44	106.4	8564.4	0.63 ± 0.03	0.54 ± 0.27
DKRL8	Pandoh, Mandi	*V. canescens*	858	5075.14	73.98	217.60	19.92	1936.18	0.72	321.56	91.7	9065.1	0.73 ± 0.06	0.66 ± 0.33
DKRL9	Batour, Mandi	*V. canescens*	940	4258.44	73.16	261.08	20.84	1892.24	3.36	412.86	104.2	10130	0.53 ± 0.03	0.33 ± 0.17
DKRL10	Chauntra, Mandi	*V. canescens*	1220	4462.04	74.32	381.48	20.84	1901.00	2.60	359.14	111.5	9146.3	1.23 ± 0.15	0.39 ± 0.20
DKRL11	Kamand, Mandi	*V. pilosa*	1269	4241.42	51.56	287.34	16.68	1895.64	1.48	390.52	64.9	14495	0.64 ± 0.02	0.92 ± 0.46
DKRL12	Kullu	*V. canescens*	1279	1824.6	50.88	203.32	26.40	1840.68	2.22	534.06	137.3	2301.5	0.81 ± 0.01	1.09 ± 0.55
DKRL13	Chandpur, Kangra	*V. canescens*	1482	4647.82	75.50	322.96	23.16	1858.34	10.2	410.34	97.0	13433	0.49 ± 0.01	0.23 ± 0.12
DKRL14	Gulera, Chamba	*V. canescens*	1639	1353.16	116.18	116.54	25.48	1854.10	6.02	360.32	54.9	8621.5	0.59 ± 0.02	0.67 ± 0.34
DKRL15	Barot, Mandi	*V. pilosa*	1829	5145.58	57.42	239.88	18.06	1841.24	2.60	370.02	94.5	11318	0.68 ± 0.06	0.44 ± 0.22

*n* = 3, Hg, Cd, As and Pb were absent in all the samples TPC—total phenolic content; TFC—total flavonoid content.

**Table 2:** Phenolic compounds present in different locations of Viola species.

Sample Code	Altitudes (Meters)	Species	Polyphenols (mg/g), Rt									
			Vanillic Acid (5.74)	Syringic Acid (6.09)	Caffeic Acid (6.45)	Epicatechin (6.63)	*p*-Coumaric acid (8.21)	Ferulic Acid (8.40)	Rutin (9.05)	Quercetin (11.95)	Luteolin (11.998)	Total
DKRV1	375	*V. canescens*	NQ	NQ	0.00 ± 0.00	0.13 ± 0.01	15.67 ± 0.99	0.03 ± 0.001	0.27 ± 0.02	ND	0.11 ± 0.01	16.24 ± 1.03
DKRV2	478	*V. canescens*	NQ	NQ	NQ	0.11 ± 0.00	2.06 ± 0.08	0.02 ± 0.001	0.26 ± 0.02	ND	0.09 ± 0.00	2.54 ± 0.10
DKRV3	492	*V. canescens*	NQ	NQ	ND	0.04 ± 0.002	12.13 ± 0.66	0.02 ± 0.00	0.47 ± 0.01	ND	0.09 ± 0.00	12.73 ± 0.672
DKRV4	699	*V. canescens*	0.3 ± 0.01	ND	ND	0.08 ± 0.003	14.29 ± 1.2	0.02 ± 0.001	0.37 ± 0.02	ND	0.10 ± 0.00	14.84 ± 1.234
DKRV5	782	*V. canescens*	ND	NQ	ND	0.04 ± 0.001	10.38 ± 0.76	ND	0.19 ± 0.01	ND	ND	10.61 ± 0.771
DKRV6	787	*V. canescens*	ND	NQ	0.05 ± 0.002	0.29 ± 0.013	15.39 ± 1.11	0.01 ± 0.00	0.39 ± 0.00	ND	0.01 ± 0.00	16.14 ± 1.125
DKRV7	793	*V. canescens*	0.01 ± 0.001	ND	0.10 ± 0.003	0.29 ± 0.015	19.25 ± 1.23	ND	0.49 ± 0.03	ND	ND	20.12 ± 1.27
DKRV8	858	*V. canescens*	0.04 ± 0.002	ND	ND	0.18 ± 0.012	20.28 ± 1.66	ND	0.59 ± 0.02	ND	ND	21.05 ± 1.694
DKRV9	940	*V. canescens*	0.03 ± 0.001	ND	0.02 ± 0.001	0.13 ± 0.01	13.90 ± 0.88	0.01 ± 0.00	0.24 ± 0.01	ND	ND	14.30 ± 0.902
DKRV10	1220	*V. canescens*	0.06 ± 0.0021	ND	0.01 ± 0.0	0.04 ± 0.00	5.51 ± 0.05	0.10 ± 0.01	0.65 ± 0.05	ND	ND	6.30 ± 0.112
DKRV11	1269	*V. pilosa*	0.05 ± 0.00	NQ	0.01 ± 0.0	ND	17.18 ± 1.33	ND	0.51 ± 0.01	ND	ND	17.71 ± 1.34
DKRV12	1279	*V. canescens*	0.05 ± 0.002	0.02 ± 0.00	ND	ND	6.49 ± 0.43	ND	4.55 ± 0.04	0.02 ± 0.0	0.21 ± 0.01	11.28 ± 0.482
DKRV13	1482	*V. canescens*	0.03 ± 0.001	ND	0.05 ± 0.003	0.18 ± 0.007	16.61 ± 1.21	0.01 ± 0.00	0.47 ± 0.01	ND	ND	17.32 ± 1.231
DKRV14	1639	*V. canescens*	0.04 ± 0.001	ND	0.01 ± 0.001	0.05 ± 0.00	23.41 ± 1.77	ND	0.54 ± 0.01	ND	ND	24.01 ± 1.782
DKRV15	1829	*V. pilosa*	0.01 ± 0.00	ND	0.04 ± 0.002	0.25 ± 0.01	5.02 ± 0.05	ND	4.68 ± 0.00	ND	ND	9.99 ± 0.062
DKRL1	375	*V. canescens*	0.01 ± 0.001	0.04 ± 0.003	ND	ND	ND	4.91 ± 0.06	1.12 ± 0.09	ND	0.06 ± 0.001	6.16 ± 0.155
DKRL2	478	*V. canescens*	0.01 ± 0.00	0.02 ± 0.001	ND	ND	ND	11.68 ± 1.1	0.40 ± 0.02	ND	0.04 ± 0.003	12.15 ± 1.124
DKRL3	492	*V. canescens*	0.04 ± 0.001	0.01 ± 0.001	NQ	ND	ND	5.56 ± 0.34	0.38 ± 0.01	ND	0.04 ± 0.004	6.02 ± 0.356
DKRL4	699	*V. canescens*	ND	0.03 ± 0.002	ND	ND	ND	11.12 ± 0.8	0.99 ± 0.04	ND	0.09 ± 0.002	12.21 ± 0.844
DKRL5	782	*V. canescens*	0.03 ± 0.00	0.05 ± 0.004	NQ	0.07 ± 0.002	ND	8.99 ± 0.65	4.21 ± 0.11	ND	0.23 ± 0.00	13.58 ± 0.766
DKRL6	787	*V. canescens*	0.05 ± 0.00	0.01 ± 0.00	NQ	0.01 ± 0.00	0.08 ± 0.002	7.10 ± 0.33	0.97 ± 0.06	ND	0.16 ± 0.01	8.36 ± 0.402
DKRL7	793	*V. canescens*	ND	0.03 ± 0.001	ND	0.09 ± 0.005	0.13 ± 0.06	8.36 ± 0.23	2.85 ± 0.09	ND	0.21 ± 0.02	11.66 ± 0.406
DKRL8	858	*V. canescens*	0.08 ± 0.001	0.05 ± 0.003	ND	0.05 ± 0.003	0.15 ± 0.01	8.40 ± 0.22	0.75 ± 0.03	ND	0.05 ± 0.00	9.53 ± 0.267
DKRL9	940	*V. canescens*	0.01 ± 0.00	0.12 ± 0.001	ND	0.13 ± 0.01	0.82 ± 0.06	6.61 ± 0.54	3.46 ± 0.14	ND	0.27 ± 0.01	11.41 ± 0.761
DKRL10	1220	*V. canescens*	0.04 ± 0.001	0.03 ± 0.002	0.09 ± 0.005	0.06 ± 0.002	1.62 ± 0.09	8.55 ± 0.66	10.63 ± 0.7	ND	0.34 ± 0.03	21.37 ± 1.49
DKRL11	1269	*V. pilosa*	0.06 ± 0.003	0.02 ± 0.001	ND	0.01 ± 0.00	ND	3.32 ± 0.21	0.31 ± 0.01	ND	0.25 ± 0.02	3.96 ± 0.244
DKRL12	1279	*V. canescens*	0.03 ± 0.002	ND	ND	ND	ND	1.78 ± 0.05	0.23 ± 0.00	ND	0.14 ± 0.00	2.18 ± 0.052
DKRL13	1482	*V. canescens*	ND	0.09 ± 0.004	0.02 ± 0.003	ND	0.33 ± 0.02	14.97 ± 1.2	1.42 ± 0.05	ND	0.43 ± 0.03	17.29 ± 1.307
DKRL14	1639	*V. canescens*	ND	0.01 ± 0.00	NQ	ND	ND	3.52 ± 0.15	0.46 ± 0.04	ND	0.26 ± 0.01	4.25 ± 0.2
DKRL15	1829	*V. pilosa*	ND	0.02 ± 0.001	NQ	0.01 ± 0.00	ND	2.41 ± 0.11	1.62 ± 0.01	ND	0.31 ± 0.01	4.36 ± 0.131

*n* = 3; ND: not detected; NQ: not quantifiable; Rt: Retention time.

**Table 3:** Antimicrobial screening of *Viola* species (10 mg/mL) against bacterial strains and MICs of the most effective plant extracts against Gram-positive bacterium, i.e., *S. aureus* MTCC96 and *B. subtilis* MTCC121.

		Zone of Inhibition in Radius (mm)															
	**Flowers**	DKRV1	DKRV2	DKRV3	DKRV4	DKRV5	DKRV6	DKRV7	DKRV8	DKRV9	DKRV10	DKRV11	DKRV12	DKRV13	DKRV14	DKRV15 a	DKRV15 b
	Species	*V. canescens*	*V. canescens*	*V. canescens*	*V. canescens*	*V. canescens*	*V. canescens*	*V. canescens*	*V. canescens*	*V. canescens*	*V. canescens*	*V. pilosa*	*V. canescens*	*V. canescens*	*V. canescens*	*V. pilosa*	*V. pilosa*
	Altitudes	375	478	492	699	722	787	793	858	940	1220	1269	1279	1482	1639	1829	
Gram +ve bacteria	*B. subtilis* MTCC121	0	1.25	1.25	1.5	2.25	2.25	5.25	4.125	4.75	3.25	2.25	5	5	4.25	4.25	0
	*S. aures* MTCC96	0	0	0	0	1	1	1	1.25	2.25	2.25	1.75	2.5	2.5	3.25	3.25	0
Gram -ve bacteria	*S. typhimurium* MTCC733	0	0	0	0	0	0	0	0	0	0	0	0	0	0	0	0
	*E. coli* MTCC43	0	0	0	0	0	0	0	0	0	0	0	0	0	0	0	0
	**Leaves**	DKRL1	DKRL2	DKRL3	DKRL4	DKRL5	DKRL6	DKRL7	DKRL8	DKRL9	DKRL10	DKRL11	DKRL12	DKRL13	DKRL14	DKRL115 a	DKRL15 b
	Species	*V. canescens*	*V. canescens*	*V. canescens*	*V. canescens*	*V. canescens*	*V. canescens*	*V. canescens*	*V. canescens*	*V. canescens*	*V. canescens*	*V. pilosa*	*V. canescens*	*V. canescens*	*V. canescens*	*V. pilosa*	*V. pilosa*
Gram + ve bacteria	*B. subtilis* MTCC121	4	2	3	2.5	2.5	3	3	3	3.5	2	2.5	3	4	4	2	2.5
	*S. aures* MTCC96	3	2	3	2.5	2.5	3	3	3	3.5	2	2.5	3	4	4	2	2.5
Gram − ve bacteria	*S. typhimurium* MTCC733	0	0	0	0	0	0	0	0	0	0	0	0	0	0	0	0
	*E. coli* MTCC43	0	0	0	0	0	0	0	0	0	0	0	0	0	0	0	0
**Minimum inhibitory concentrations (mm) against Gram + bacteria**																	
	**Samples**	DKRL1		DKRL7		DKRL13		DKRL14		DKRV7		DKRV9		DKRV12		DKRV13	
	Species	*V. canescens*		*V. canescens*		*V. canescens*		*V. canescens*		*V. canescens*		*V. canescens*		*V. canescens*		*V. canescens*	
Amount	Altitudes	375		793		1482		1639		793		940		1482		1639	
5 mg	*B. subtilis* MTCC121	1.25 ± 0.35		2.0 ± 0.0		1.25 ± 0.35		1.25 ± 0.35		2.25 ± 0.35		2.12 ± 0.18		3.25 ± 0.35		1.25 ± 0.35	
	*S. aures* MTCC96	1.25 ± 0.35		1.37 ± 0.18		1.25 ± 0.35		2.25 ± 0.35		0.5 ± 0.0		1.25 ± 0.35		1.5 ± 0.71		0.75 ± 0.35	
6 mg	*B. subtilis* MTCC121	4.5 ± 0.71		3.0 ± 0.0		4.25 ± 0.35		4.25 ± 0.35		5.25 ± 0.35		5.25 ± 0.0		4.75 ± 0.35		5.12 ± 0.18	
	*S. aures* MTCC96	3.0 ± 0.0		3.0 ± 0.0		4.25 ± 0.35		4.25 ± 0.35		1.25 ± 0.35		2.25 ± 0.0		2.5 ± 0.0		2.5 ± 0.0	

## Data Availability

Not applicable.

## Supplementary Materials

The following supporting information can be downloaded at: https://www.mdpi.com/article/10.3390/molecules27123867/s1, Table S1. Voila species study area; Figure S1. Micro-nutrients in Viola species at gradient altitudes (in ppm), Figure S2. Macro-nutrients in Viola species at gradient altitudes (in ppm); Figure S3. ABTS & DPPH based antioxidant activity of (IC50 µg/mL) of Viola species; Figure S4a UPLC-DAD Chromatograms of flowers samples of Viola species (DKRL1-DKRL15); Figure S4b UPLC-DAD Chromato-grams of flowers samples of Viola species (DKRV1-DKRV15); Table S2. MIC’s of the most effective plant extract against *S. aureus* & *B. subtilis*; Figure S5. Antimicrobial activity (Zone of inhibition of flowers and leaves) of Viola species.

## Sample Availability

Samples are available from the authors.
