# Supplementary material for: Polyphenolic Profiling, Antioxidant, and Antimicrobial Activities Revealed the Quality and Adaptive Behavior of Viola Species, a Dietary Spice in the Himalayas
Source: Molecules. 2022 Jun 16;27(12):3867. doi: 10.3390/molecules27123867 (PMC9230710; doi:10.3390/molecules27123867)
Supplement: Supplementary file 1 [file molecules-27-03867-s001.zip › molecules-1653680-supplementary.pdf]

Supplementary Information

Supplementary Table S1. Voila species study area: Altitude, plant development phase, time of sampling; habitats, collection sites in Himachal Pradesh.

**Code**	**Altitude**	**Species**	**Habitat**	**Plant Phase**	**Percentage yield**	**Location of collection**	**Coordinates**
**DKR V1**	375	*Viola canescnes*	Forest-shady locations	Flowering	35.66	**Berthin, Bilaspur**	31.4188° N, 76.6427° E
**DKR V2**	478	*Viola canescnes*	Forest-shady locations	Flowering	41.08	**Telkar, Hamirpur**	31.6242° N, 76.5692° E
**DKR V3**	492	*Viola canescnes*	Forest-shady locations	Flowering	37.83	**Berru, Hamirpur**	31.6795° N, 76.5387° E
**DKR V4**	699	*Viola canescnes*	Forest-shady locations	Flowering	35.78	**Ghumarwin, Bilaspur**	31.4491° N, 76.7048° E
**DKR V5**	782	*Viola canescnes*	Road sides	Flowering	44.61	**Bijni, Mandi**	31.7460° N, 76.9441° E
**DKR V6**	787	*Viola canescnes*	Forest-shady locations	Flowering	33.11	**Chabutra, Hamirpur**	31.7699° N, 76.4888° E
**DKR V7**	793	*Viola canescnes*	High mountain pasture	Flowering	30.5	**Paddar, Mandi**	31.8794° N, 76.9160° E
**DKR V8**	858	*Viola canescnes*	Road sides	Flowering	30.24	**Pandoh, Mandi**	31.6676° N, 77.0536° E
**DKR V9**	940	*Viola canescnes*	Road sides	Flowering	36.47	**Batour, Mandi**	31.7344° N, 76.8865° E
**DKR V10**	122 0	*Viola canescnes*	High mountain pasture	Flowering	32.02	**Chauntra, Mandi**	32.0138° N, 76.7495° E
**DKR V11**	126 9	*Viola pilosa*	High mountain pasture	Flowering	28.96	**Kamand, Mandi**	31.7781° N, 76.9974° E
**DKR V12**	127 9	*Viola canescnes*	Forest-shady locations	Flowering	25.92	**Kullu**	31.9592° N, 77.1089° E

**DKR V13**	1482	*Viola canescnes*	High mountain pasture	Flowering	44.07	**Chandpur, Kangra**	32.1215° N, 76.5654° E
**DKR V14**	1639	*Viola canescnes*	High mountain pasture	Flowering	31.48	**Gulera, Chamba**	32.5527° N, 76.1260° E
**DKR V15**	1829	*Viola pilosa*	High mountain pasture	Flowering	28.95	**Barot, Mandi**	32.0410° N, 76.8402° E

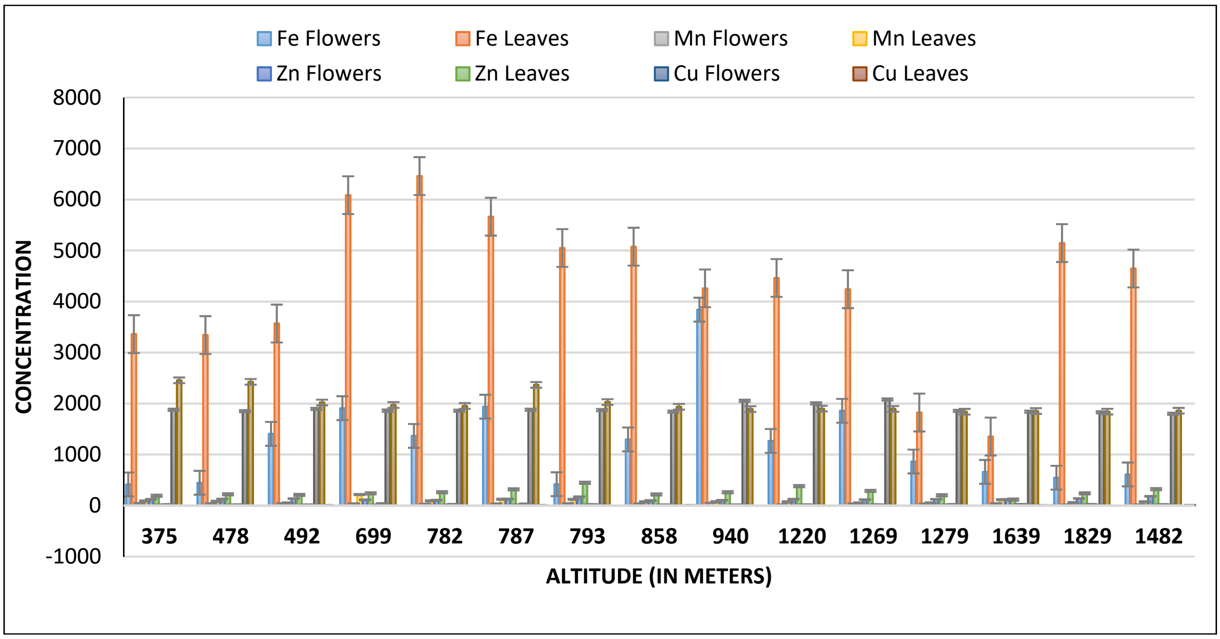

Supplementary Figure S1. Micro-nutrients in Viola species at gradient altitudes (in ppm)

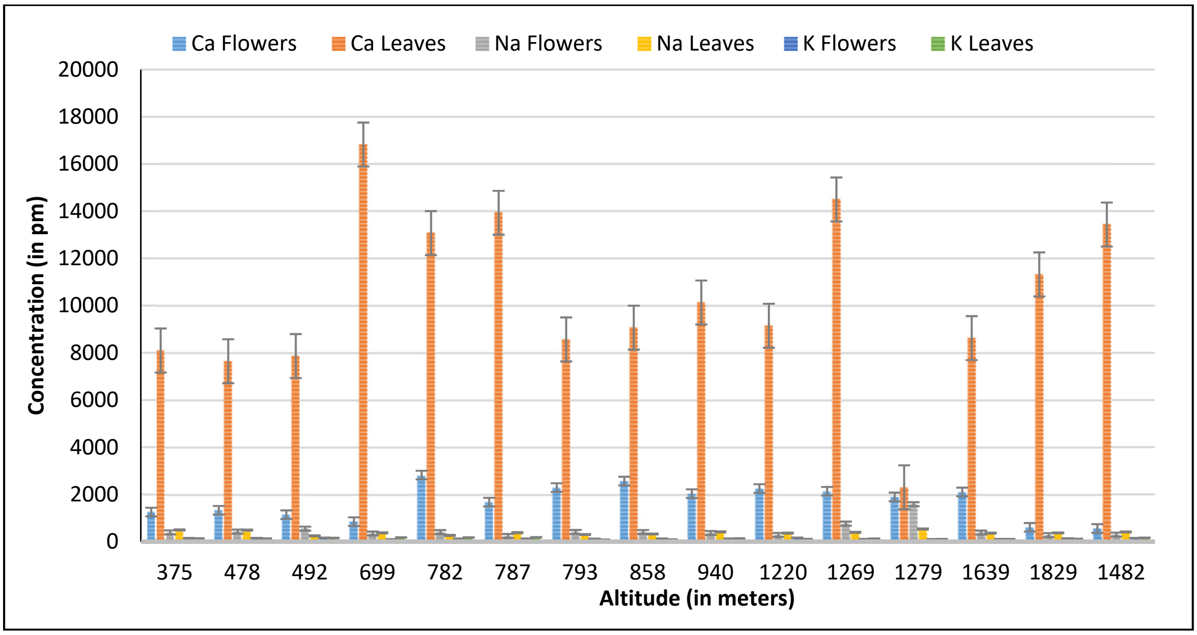

Supplementary Figure S2. Macro-nutrients in Viola species at gradient altitudes (in ppm)

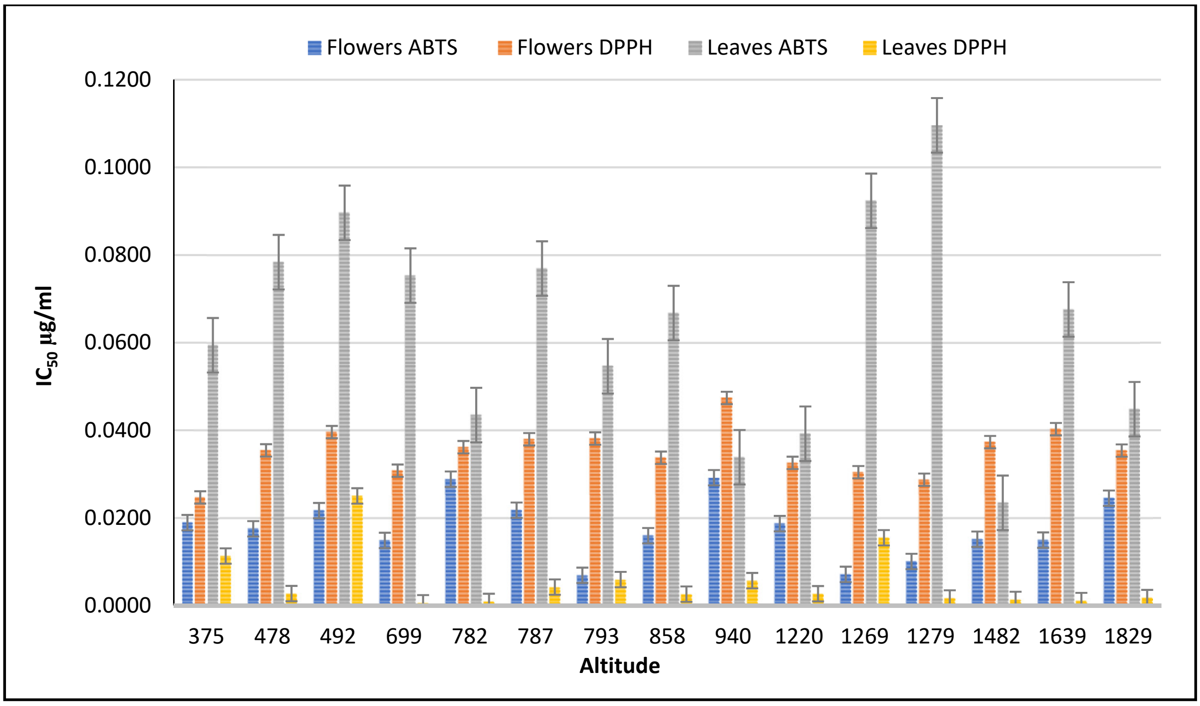

Supplementary Figure S3. ABTS & DPPH based antioxidant activity of (IC50 µg/ml) of Viola species.

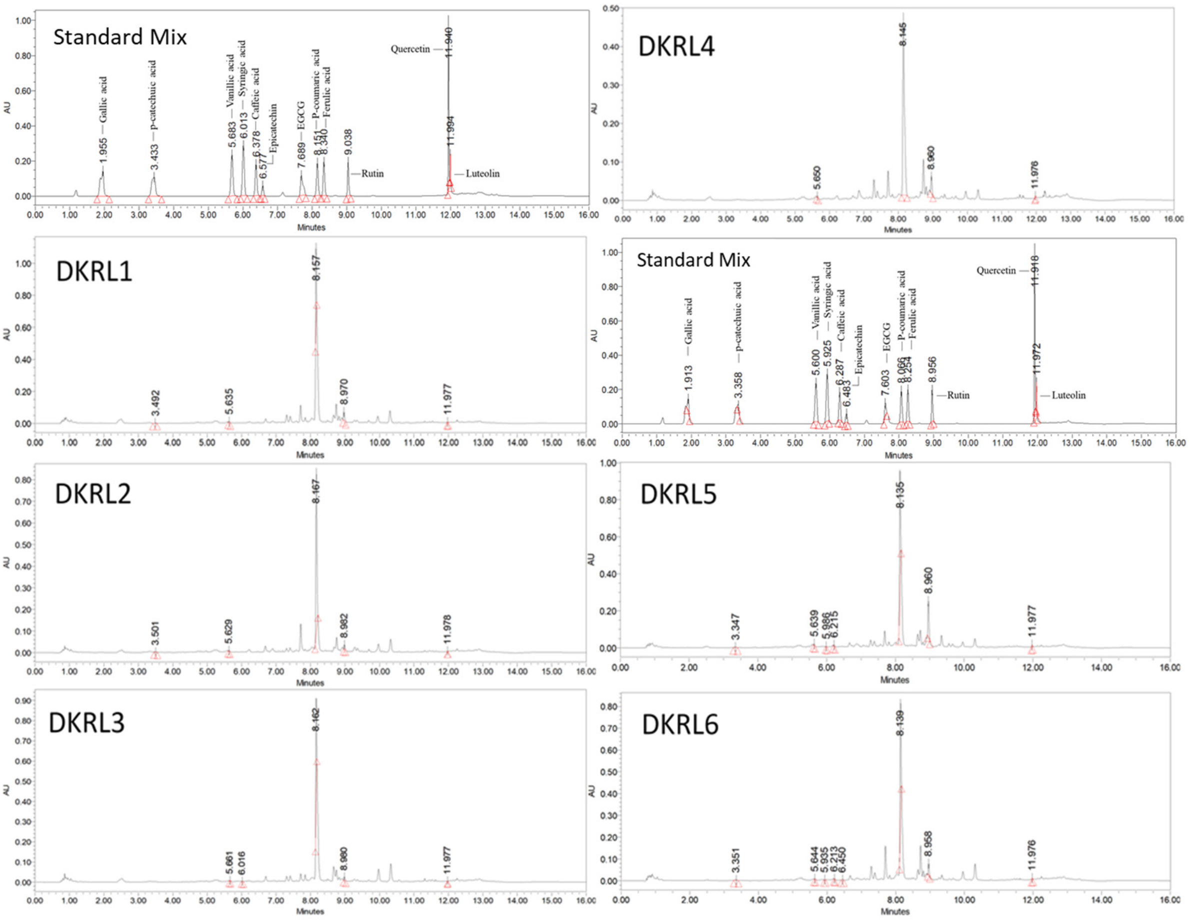

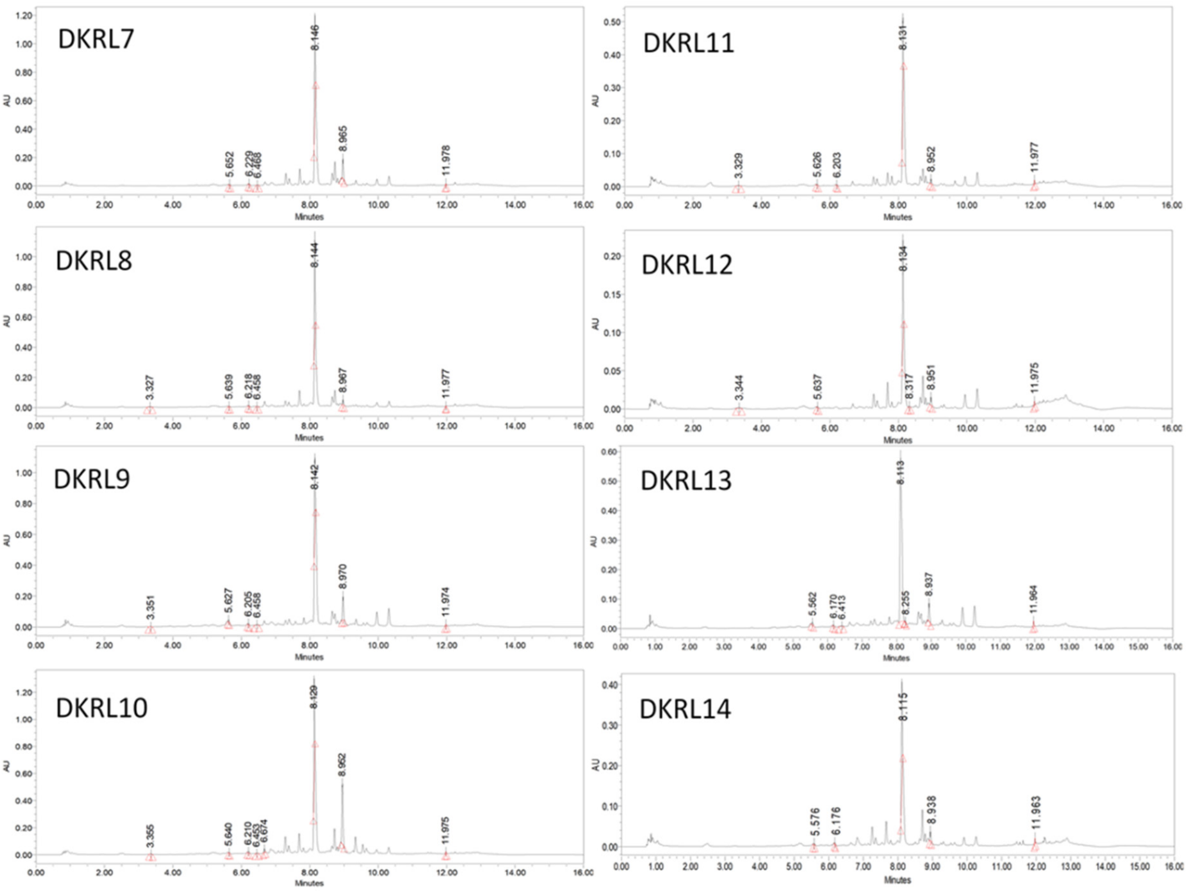

Supplementary Figure S4a. UPLC-DAD Chromatograms of flowers samples of Viola species (DKRL1-DKRL15).

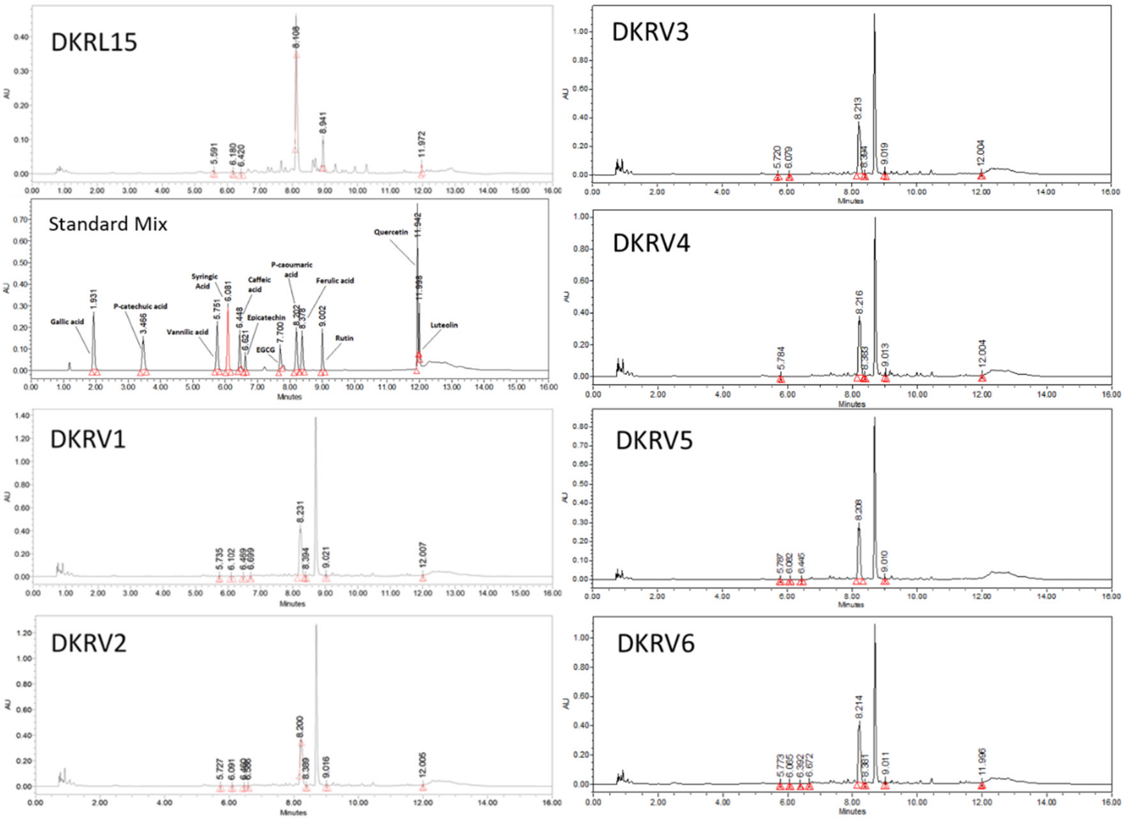

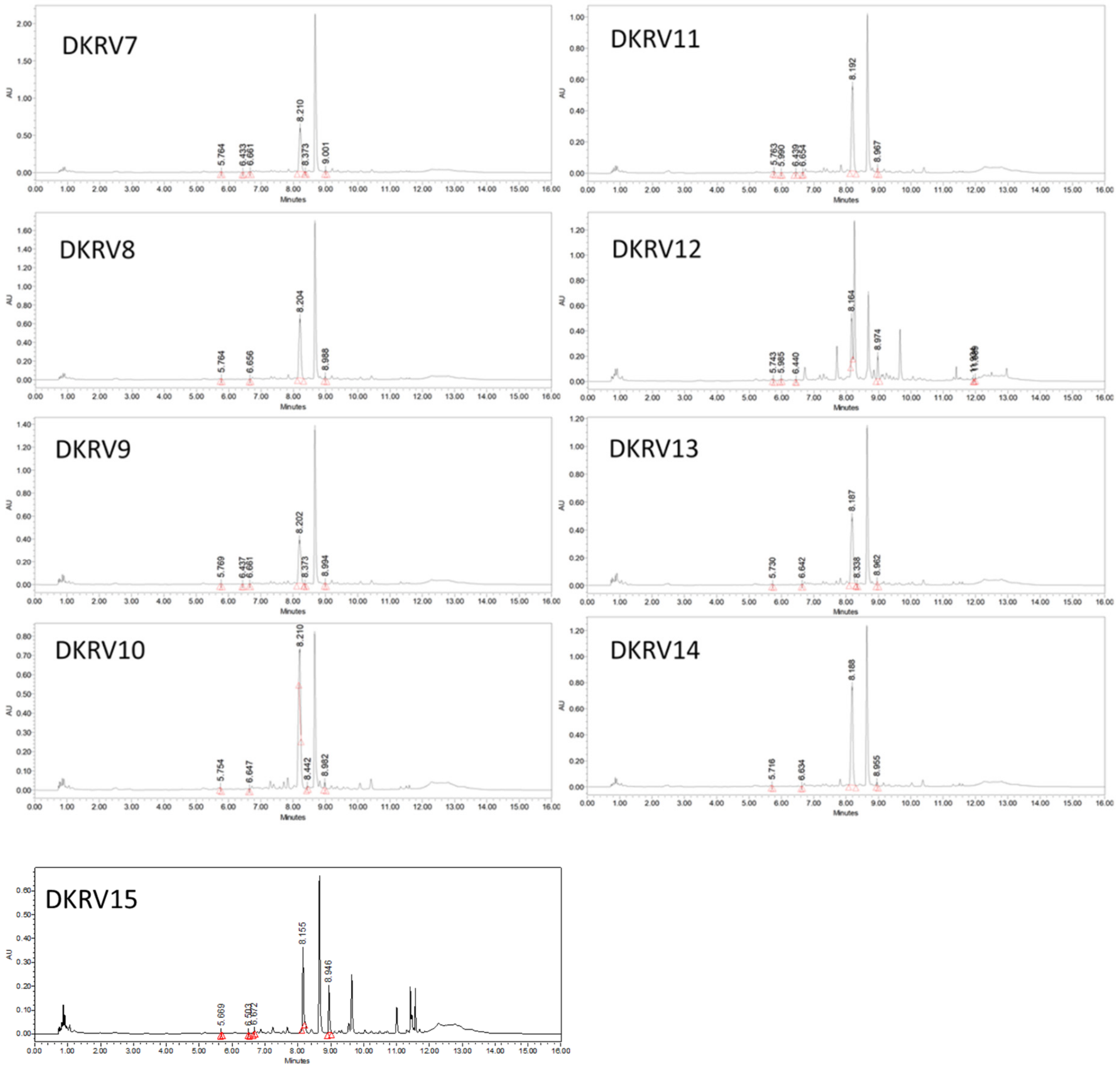

Supplementary Figure S4b. UPLC-DAD Chromatograms of flowers samples of Viola species (DKRV1-DKRV15).

Supplementary Table S2. MIC's of the most effective plant extract against *S. aureus* & *B. subtilis.*

Zone of inhibition (mm)																
Gram +ve bacteria																
	B. subtilis				*S. aureus*				*B. subtilis*				*S. aureus*			
**Conc. (mg)**	**L1**	**L7**	**L13**	**L14**	**L1**	**L7**	**L13**	**L14**	**V7**	**V9**	**V12**	**V13**	**V7**	**V9**	**V12**	**V13**
**0.5**	0	0	0	0	0	0	0	0	0	0	0	0	0	0	0	0
**1**	0	0	0	0	0	0	0	0	0	0	0	0	0	0	0	0
**2**	0	0	0	0	0	0	0	0	0	0	0	0	0	0	0	0
**3**	0	1	0	0	0	0	0	0	0	0	0	0	0	0	0	0
**4**	1	1	0	0.5	1	1	0.5	1	0	1.5	1	0.5	0	0	0	0
**5**	1.5	2	1	1	1.5	1.5	1	2	2	2	3	1	0	1	1	0.5
**6**	4	3	4	4	3	3	4	4	5	5	5	5	1	2	2.5	2.5

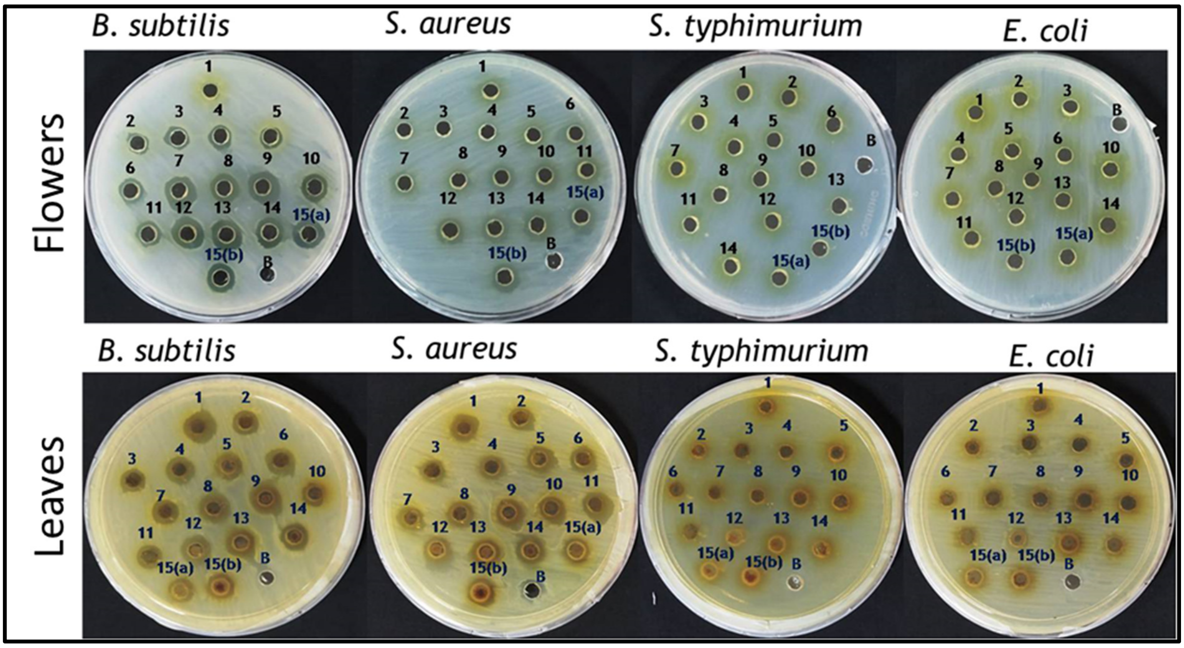

Supplementary Figure S5. Antimicrobial activity (Zone of inhibition of flowers and leaves) of Viola species.
